# Lieut.-Colonel George Herbert Rowe

**Published:** 1910-07-30

**Authors:** 


Lieut.-Colonel George Herbert Rowe has died, aged 51. Lieut.-Colonel Rowe became a member of the Royal College of Surgeons of England in 1878, and practised as a surgeon at Leeds. He was in command of the 8th Battalion of the West Yorkshire Territorials (Leeds Rifles). He was also senior house surgeon at Leeds Public Dispensary and St. Chad's Home for Waifs and Strays.

